# Improving the quality of emergency medicine care by developing a quality requirement framework: a study from The Netherlands

**DOI:** 10.1186/1865-1380-5-20

**Published:** 2012-05-23

**Authors:** David E Ikkersheim, Harm van de Pas

**Affiliations:** 1 Consultant at KPMG Plexus, Straatweg 68, Breukelen, BR 3621, The Netherlands; 2 Phd student at FALW, VU University, Amsterdam, The Netherlands; 3 Medical Director Emergency Department, St. Elisabeth Hospital, PO Box 90151, Tilburg, LC 5000, The Netherlands; 4 Medical Director Regional Ambulance Service, PO Box 3166, ‘s-Hertogenbosch, DD 5203, The Netherlands; 5 President, Netherlands Society for Emergency Physicians (NVSHA), PO Box 8003, Utrecht, RA 2503, The Netherlands

**Keywords:** Quality of care, Emergency medicine, Quality requirement framework

## Abstract

**Background:**

In The Netherlands, mainly inexperienced physicians work in the ED on all shifts, including the evening and night shifts, when no direct supervision is available. In 2004 a report of the Dutch Health Care Inspectorate revealed that quality of care at Emergency Departments (EDs) was highly variable. Based on this report and international studies showing significant potential for quality improvement, stakeholders felt the need to improve the quality of EM care. Based on the literature, a baseline measurement and a panel of experts, The Netherlands recently developed a nationwide quality requirement framework (QRF) for EM. This article describes the content of and path to this QRF.

**Methods:**

To conduct a baseline measurement, the panel needed to identify measurable entities related to EM care at EDs. This was done by formulating both qualitative and partly quantitative questions related to the following competence areas: triage system, training of personnel (physicians and nurses), facilities and supervision of physicians.

27 out of 104 Dutch EDs were sampled via a cross-sectional study design, using an online survey and standardized follow-up interview in which the answers of the survey were reviewed.

**Results:**

In the QRF, EM care is divided into a basic level of EM care and six competence certification areas (CCAs): (acute) abdominal aortic aneurysm, acute coronary syndrome, acute psychiatric behavioral disorder, cerebral vascular accident, pediatric critical care and infants with low birth weight. For the basic level of EM care and for every CCA minimum prerequisites for medical devices and training of personnel are established. The factors selected for the QRF can be regarded as minimum quality standards for EM care. A major finding of this study was that in The Netherlands, none of the 27 sampled EDs demonstrated compliance with these factors.

**Conclusion:**

Our study shows that Dutch EDs fall short of what the expert consensus panelists considered minimum prerequisites for adequate EM care. The process of systematic enquiry allowed this information to come to light for the first time, which resulted in the implementation of a QRF for Dutch ED personnel, that is intended improve quality of EM care over time. This is an important development for the worldwide EM community as the QRF shows a way to generate interim standards to improve the chances of appropriate delivery of EM care when the gold standard of providing fully qualified EPs is not initially achievable.

## Background

In the last few decades, emergency medicine (EM) has developed as a specialty at different paces in different countries. While the Dutch health care system delivers good quality care compared to some other health care systems, EM is still an evolving specialty in The Netherlands [1-4]. Since the founding of The Netherlands Society of Emergency Physicians (NVSHA) in 1999, the need for improving the quality of Dutch EM care has received more attention [5]. This growing attention led to preliminary recognition of emergency physicians (EPs) in 2008, with the possibility of recognition as a medical specialty in the future. From 1999 on EM training programs became more standardized and a separate EM residency was created in 2004, which was officially recognized in 2008.

There is currently a consensus in The Netherlands that emergency departments (EDs) should be staffed 24/7 with EPs. Nonetheless, the shortage of EPs has prevented most EDs from being fully staffed with EPs; instead, EDs are mostly staffed by physicians who recently graduated from medical school and/or medical residents who work under the supervision of medical specialists or EPs. In the Dutch system, the choice to seek supervision or advice is up to the (junior) physician who is seeing the patient. Even when supervision is requested, the patient is not always seen by the supervising specialist during the patient’s time in the ED, and supervision is often only provided by telephone. Thus, relatively inexperienced physicians working at Dutch EDs have substantial responsibilities for patient care [5,6].

In 2004 the Dutch health-care inspectorate recognized that the quality of EM care could be improved and published a report addressing the quality of EM care in The Netherlands [7]. The relevant conclusions can be summarized as follows:

· The ambitions of EDs to provide high quality EM care do not always match their actual ability to do so.

· The quality of EM care varies at each ED.

· There is a need to develop requirements for a minimum level of training and competencies for physicians who work in EDs.

· Medical devices are not always available and personnel are not always trained to treat patients according to the latest standards of care.

In addition to the specific Dutch circumstances, also international studies indicate that quality and safety of (EM) care can be improved [8-12]. In addition, studies show that a better quality of care can lower complication rates and thereby lower growth of health care costs [11]. One of the strategies to actually improve the quality and safety of (EM) care is to treat patients via standardized clinical pathways according to the best available evidence [13].

To achieve the goal of a better quality of EDs with experienced professionals who are working according to the best available evidence, the government and other stakeholders realized that apart from appropriate facilities and quality monitoring systems, it is essential to have well trained medical professionals working at EDs. Ideally this would mean that all EDs would be staffed 24/7 with EPs, but with the understanding that it will take at least 10 years to train a sufficient number of EPs to staff all Dutch EDs and thereby resolve quality issues, stakeholders in the Dutch health-care system felt the need to find interim ways to improve the quality of care at EDs [6].

**Figure 1:**
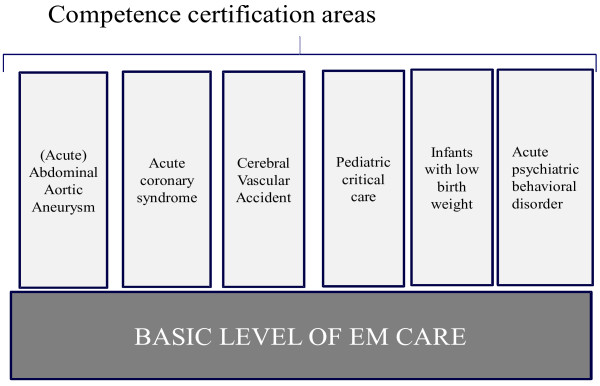
Composition of the quality requirement framework.

Accordingly, the Dutch Ministry of Health created an expert panel to develop a quality requirement framework (QRF) for basic EM care at EDs. In addition, the expert panel had to formulate basic prerequisites for more complicated acute conditions that are usually treated initially by personnel working in the ED. These were termed competence certification areas (CCAs) next to the basic level of EM care (Figure 1).^a^ In this article, we introduce the QRF, which was developed primarily via expert panel consensus and by reviewing current guidelines, and which was designed to provide the minimum prerequisites necessary for acceptable basic level EM care. In addition, we report the baseline performance of Dutch EDs with regard to the factors identified as the minimum prerequisites for basic EM care.

## Methods

The Ministry of Health first assembled a panel of 20 experts that consisted of stakeholders (mostly non-EPs but rather stakeholders such as trauma surgeons, ambulance personnel, nurses, etc.) that could formulate a QRF.^b^ The panel began by conducting a literature search for existing QRFs related to the basic level of EM care that might be useful for addressing the Dutch situation. Although some studies regarding EM quality requirements and performance indicators were found, none were regarded by the expert panel as completely applicable to the specific situation of EM care as an evolving specialty in The Netherlands [14-17]. The panel then realized that a baseline measurement was needed to describe current practices, to select the most appropriate factors to include in the QRF, and to evaluate the impact of the QRF over time. To determine appropriate CCAs, the panel reviewed guidelines from Dutch scientific associations and studied current practices to determine the minimum standards for CCAs in terms of personnel training and facilities (medical devices, available infrastructure) [18-20]. Six CCAs were selected based on the priorities and judgment of the expert panel.

### Baseline measurement: questionnaire and follow-up interview

To conduct a baseline measurement, the panel needed to identify measurable entities related to EM care at EDs. This was done by formulating both qualitative and partly quantitative questions related to the following competence areas: triage system, training of personnel (physicians and nurses), facilities and supervision of physicians.

EDs were sampled via a cross-sectional study design, using an online survey and standardized follow-up interview in which the answers of the survey were reviewed. This resulted in a physician-created survey comprised of 120 mostly multiple-choice questions that were filled out by the medical and/or managerial heads of the EDs. The application ‘SurveyMonkey’ was used for the survey, and answers were recorded in Microsoft Excel version 2007. To encourage truthful answers, anonymity in the final report that was presented to the stakeholders was guaranteed for the individual EDs that filled out the questionnaire. Examples of the questions are shown in Table 1.

**Table 1:** Examples of questions used for baseline measurement

**Area of competence**	**Examples of questions out of baseline measurement**
**Basic level of EM care: physicians**	Is there a compulsory training program in place for physicians who start working in your ED?
**Basic level of EM care: nurses**	Do ED nurses in training also work night shifts without supervision?
**CCA: cerebral vascular accidents**	Is there a 24/7 availability of a thrombolysis team?
**CCA: (acute) abdominal aortic aneurysm**	Is there always a vascular surgeon available to conduct (acute) abdominal aortic aneurysm surgery during evening, weekend and night shifts?

An onsite interview to review the answers was subsequently conducted with two researchers, one of them being a physician who was present at all interviews. The interviews were held with the medical/or managerial heads of the ED who had previously filled in the questionnaire. The interviewers did not receive special training in conducting structured interview techniques. As each answer was reviewed one by one, any corrections to the answers were typed in during the interview and checked with the interviewees. The baseline measurement was conducted from March to June 2009, and the QRF was established in December 2009.

### Participating hospitals

Currently, there are 92 hospitals in The Netherlands. Most have one ED, but several have two locations with EDs. In total there are 104 EDs [6]. A requirement of the baseline measurement was that it be representative of all 104 EDs in The Netherlands. The final baseline measurement included a total of 27 EDs in three different regions across the country, including both rural and urban and large and small EDs, which were associated with different types of hospitals (academic, large peripheral teaching hospitals and general hospitals). The sample of 27 EDs is compared to the nationwide average representative in terms of type of hospital, as displayed in Table 2. As 52 of 104 (50%) nationwide EDs are located in rural areas, the rural EDs are slightly overrepresented (16 out of 27 or 60%) [21].

**Table 2:** Participating hospitals

**Regions**	**Region 1 (urban)**	**Region 2 (rural)**	**Region 3 (rural)**	**Total within sample (27 hospitals)**	**Nationwide 92 hospitals**
**Number of EDs**	11	12	4	27	104
**Average of patient visits per ED per year (2008)**	25,291	13,948	18,217	19,202	unknown
**(min-max)**	(3,870-48,000)	(3,466-23,000)	(11,365-27,000)	(3,466-48,000)	
**Academic hospitals**	1	1	0	2/27 (7%)	8/92 (9%)
**Large peripheral teaching hospitals**	5	2	1	8/27 (30%)	26/92 (28%)
**General hospitals**	5	9	3	17/27 (63%)	58/92 (63%)

The baseline measurement provided an overview of current ED practices regarding the following: triage system, training of personnel (physicians and nurses), facilities and physician supervision. These results were presented to the expert panel. The criteria that the expert panel used to select quality requirements were:

· Differentiation between EDs: if all EDs in the sample already met the quality requirement, it was not included in the QRF.

· Feasibility for the majority of EDs: the majority of EDs should be able to reach compliance within a 1-year period (only for the basic level of care) according to the ED management and expert panel (Table 3) [17].

**Table 3:** Selection of quality requirements

**Requirement**	**Differentiation: 100% compliance found?**	**Feasible to implement within 1 year?**
**Triage system available and trained personnel that can conduct proper triage**	Yes	n/a
**Trained personnel available 24/7 physicians: Advanced Life Support, ABCDE, Advanced Trauma Life Support or in hospital training program**	No	Yes
**Trained personnel available 24/7 Nurses: Trauma Nursing Core Course, Emergency Nursing Pediatric Course or in hospital training program**	No	Yes
**Facilities (X-ray, echo, ECG, resuscitation equipment, direct lab availability)**	Yes	n/a
**Supervision of medical specialist 24/7 available and formalized in consent**	Yes	n/a

### Costs of QRF

Lastly we calculated the costs of implementing the QRF for hospitals. Per requirement we calculated the costs of implementation using data regarding the nationwide number of physicians and nurses working on EDs multiplied by the costs of a specific training, using the Dutch prices of the international certified courses (for requirements in rows 2, 4, 5 and 6 in Table 4) [6]. For the requirements in rows 1, 3 and 7 we calculated the labor costs of junior physicians working in EDs (requirements in row 1 and 7) and the costs of supervising EPs or medical specialists (requirement in row 3). If a prerequisite was already met at some point in time during the physician or nurse working in the ED, no costs were included as rescheduling of the training would then be sufficient.

**Table 4:** Results of baseline measurement of the basic level of EM care and costs for implementation

	**Number of EDs that meet the requirement before start of employment for ED physicians or nurses**	**Number of EDs that meet the requirement at some point in time during physician or nurse working in ED**	**Estimated costs of implementation per year nationwide (first year of introduction)**
**1. A training program in which the physician works supernumerary**	23/27 (85%)	n/a	€ 374, 000- € 561,000
**2. Advanced Life Support training for physicians working on the ED as part of the training program**	14/27 (52%)	22/27 (82%)	€ 43,000 – € 65,000
**3. Evaluation conversation between head of ED and new physician after the training program in which the taught competencies are discussed**	17/27 (63%)	n/a	€ 64,000 – € 96,600
**4. During the training program training in the ABCDE systematic, comparable to the level of the Advanced Trauma Life Support®**	0/27 (0%)	13/27 (48%)	€ 6,100,000 - € 11,300,000
**5. Per shift availability of one nurse with specific training in trauma nursing, comparable to the level of the Trauma Nursing Core Course®**^**a**^	12/27 (44%)	19/27 (69%)	€ 113,000 - € 226,000
**6. Per shift availability of one nurse with specific training in pediatric nursing, comparable to the level of the Emergency Nurse Pediatric Course®**	2/27 (7%)	10/27 (37%)	€ 230,000 - € 460,000
**7. Doctor present at the ED during opening times of the ED**	26/27 (96%)	n/a	€ 0 - € 830,000
		**n/a = not applicable**	**Total: € 7 million - € 14 million**

^a^Although this requirement applies to the ED and not to individuals, many EDs have only one (specialized) nurse scheduled per night shift, resulting in the situation that if the nurse meets the requirement the ED also does. Therefore, we display the percentages of both the EDs that comply before the start of employment (mostly EDs with multiple (specialized) nurses per night shift) and the percentage of EDs that meet the requirement at some point in time during the nurse working in the ED.

## Results

Table 4 shows only the results of the baseline measurement test that concerned the training of personnel (physicians and nurses). All EDs showed full compliance in the other competence areas (triage system, facilities, supervision), as shown in Table 3. None of the 27 sampled EDs met the (minimum) standards of the quality requirements that were identified by the expert panel regarding the training of personnel. In addition, we present the annual costs per requirement to achieve nationwide compliance for all EDs in the first year of implementation.

As all elements of Table 4 were regarded as minimum prerequisites for providing quality EM care, the expert panel decided to translate the findings shown in Table 4 into the QRF (Table 5). The panel gave the recommendation that all Dutch EDs should comply to this QRF within 1 year.

**Table 5:** The quality requirement framework

	**Requirements**
**Basic level of care: physicians**	· A training program in which the physician works supernumerary in which competencies given below are taught and tested^a^
	· During the training program a training in the ABCDE systematic, comparable to the level of the Advanced Trauma Life Support® training is required
	· At all times, the ED should be able to have a physician who is trained in resuscitation (ALS or training provided by hospital^b^) and intubation within 5 min at the bed of the patient
**Basic level of care: nurses**	· Per shift availability of one nurse with specific training in triage
	· Per shift availability of one nurse with specific training in trauma nursing, comparable to the level of the Trauma Nursing Core Course®
	· Per shift availability of one nurse with specific training in pediatric nursing, comparable to the level of the Emergency Nurse Pediatric Course®
**(Acute) Abdominal aortic aneurysm (AAA)**	Indication:
	· Clinical suspicion of (acute) abdominal aortic aneurysm
	Facilities:
	· Direct availability of vascular surgeon· Direct availability of CT scan
	· Availability of endovascular stenting procedure in the hospital
	· Presence of intensive care.
**Acute coronary syndrome (ACS)**	Indications:
	· Patients with acute coronary syndrome and ST elevation on the electrocardiogram (ECG)
	· Patients with acute coronary syndrome without ST elevation on the ECG, but with other indications for PCI such as NYHA-4, diabetes mellitus, hemodynamic instability
	Facilities:
	· Direct availability of interventional cardiologist
	· Cardiac catheterization facilities: fractional flow reserve, intravascular ultrasound, defibrillation, balloon pump, ablation technique, resynchronization therapy
**Acute psychiatric behavioral disorder**	Indication:
	· Patients with an (acute) behavioral disorder possibly due to intoxication, suicidality or psychotic condition
	Facilities:
	· Direct availability of psychiatrist and psychiatric nurse.
	· A room at the ED, which is suited to treat confused patients and to conduct clinical investigation
	· Availability of a psychiatric department in hospital
**Cerebral vascular accident (CVA)**	Indication:
	· Acute CVA (hemorrhagic and non-hemorrhagic)
	Facilities:
	· Direct availability of neurologist
	· Direct availability of CT scan
	· Nursing team familiar with thrombolysis procedure
**Pediatric critical care**	Indication:
	· Severely ill children
	Facilities:
	· Direct availability of pediatrician
	· Residents have had training in treating children in need of intensive care comparable to the level of Pediatric Advanced Life Support® training
	· Presence of pediatric intensive care unit
**Infants with low birth weight**	Indication:
	· Imminent birth with gestational age under 32 weeks and or a birth weight less than 1,250 g
	Facilities:
	· Direct availability of gynecologist and pediatrician
	· Neonatal intensive care unit

^a^No specific time length for the training program is defined.

^b^Training by hospital usually has a duration of 2–4 h and is often not standardized.

## Discussion

The factors selected for the QRF can be regarded as minimum quality standards for EM care. For instance, the now compulsory ABCDE training for relatively inexperienced physicians is essential for appropriate care at EDs as these physicians may treat acutely ill patients. A major finding of this study was that, in The Netherlands, none of the 27 sampled EDs demonstrated compliance with these factors. This was surprising since the Dutch health-care system is perceived as one that delivers good quality care compared to that in other countries [1-4]. Based on our findings, other countries may wish to survey their EDs as well, as overall health-care system performance and the actual compliance to quality standards for EM care may not be in concordance with each other.

Previous studies demonstrated that patient safety can be compromised in EDs, especially during evenings, weekends and night shifts, because of the double jeopardy of recently graduated physicians who are likely to have both little experience plus limited supervision [10,22]. In The Netherlands, mainly inexperienced physicians work in the ED on all shifts, including the evening and night shifts when no direct supervision is available. The QRF seeks to improve this situation, establishing minimum standards prerequisite for providing quality care, because without training in the ABCDE approach to patient management, an inexperienced physician is unlikely to be able to provide even temporary stabilizing care to potentially acutely ill patients.

The QRF is an important example approach for other EM communities as it shows a way to generate interim standards. This is one strategy to improve the chances of appropriate delivery of EM care when the gold standard of providing fully qualified EPs is not initially achievable. Following the development path of this QRF may help other countries in which EM is still an evolving specialty to develop a QRF suitable for their situations. Especially in the 12 European countries in which EM is not yet recognized as an independent specialty, this type of QRF can provide a minimum standard. At the same time, it can function as a stimulus for countries to recognize EM as an independent specialty, as countries can refer to this QRF and compare it with their own situations at EDs. If the presented minimum quality standards in this QRF are not in place yet, this may be an extra argument to position EM as an independent specialty to spur quality improvement. By creating an independent EM specialty, EDs can more explicitly focus on how to deliver and monitor appropriate EM care, instead of viewing EDs as one of the places where specialists treat their patients [23].

### Implementation of the QRF in The Netherlands

The presented consensus-based process for QRF development is typical for the Dutch consensus culture. This approach has both disadvantages and advantages. A disadvantage of a consensus-based approach is that it is time consuming: there is a 6-year period between the first report of the Inspectorate regarding EM care and the actual formulation of the QRF. In addition, the consensus approach may lead to compromises on issues that hinder further improvement of quality of EM care. For instance, the QRF decision that hospitals can self-determine training programs (such as ALS training) instead of making internationally certified ALS training compulsory could be regarded as such an issue.^c^

On the other hand, for now there seems to be broad support for the QRF within the Dutch EM care community, especially because many stakeholders (including the relevant associations of providers and professionals) were represented in the expert panel. We therefore believe that the implementation of the QRF can be successful (meaning full compliance of EDs) as long as the Inspectorate closely monitors actual implementation of hospitals. It is also worth noting that the Inspectorate embraced the QRF and made it compulsory for EDs from 2011 on. The Ministry of Health also requested the Inspectorate to conduct an evaluation 2 years after the QRF had been introduced to evaluate its impact. Recently, the results of the first 2011 initial assessment of the Inspectorate, which visited 33 EDs randomly (thus different from the sample in our study), were published. This assessment showed that 5 out of 33 EDs did fully comply with the QRF. The 28 EDs that did not fully comply were given 6 weeks time to comply with the standards of the QRF. After this time, the Inspectorate made a repeat visit. These visits showed that 27 EDs showed full compliance to the QRF and that one ED was not able to meet the standards of the QRF. This ED has been forced to shorten its ED opening hours to comply with the QRF [24].

Surprisingly, the presentation of the QRF, the baseline measurement and also the recent assessment of the Inspectorate did not receive much attention in public debate, although one newspaper article stated that this study revealed that the quality of EDs can be substantially improved [25].

### The availability versus quality tradeoff

In addition, the requirements may promote the concentration of EM care as a higher volume of patients is needed to fund training of personnel following the prerequisites. Particularly for the CCAs (such as ACS and CVA) there is a growing body of literature that shows that there is a positive ‘volume outcome’ association [26-28]. Following this, concentration of care, at least for CCAs with a positive volume outcome association, seems desirable. This may also result in a concentration of EDs, but this is not necessarily the case, as most of the volume of care at EDs does not come from CCA patients.

On the other hand, accessibility of care is important; in The Netherlands a law states that every citizen should have an ED within 45 min travel distance. But following this 45 min norm, calculations show that The Netherlands would need only 45 EDs, assuming that EDs were spread optimally over the country [29]. Hospitals, however, are very reluctant to abandon their EDs as significant quantities of patients enter the hospital via the ED. The societal debate regarding the appropriate number of EDs has recently resulted in a national agreement among hospitals, insurers and the government, aiming at a significant reduction of the numbers of EDs in The Netherlands. According to this agreement, hospitals that close their EDs will receive some financial compensation from a national fund [30].

### Cost-effectiveness

We estimated that the additional costs of implementing this QRF for the basic level of EM care, excluding the CCAs, for all 104 Dutch EDs will total 7–14 million euros in the first year [6]. After the first year, the yearly cost will be between 3 and 8 million euros per year. Compared to the annual cost of hospital health care in The Netherlands, which is 17 billion euros, the cost seems relatively small. When the societal costs of inadequately trained physicians are considered, the return on society’s investment in training is likely to be substantial [6,11].

### Limitations

The developmental process of creating the QRF presented here may have suffered from several limitations. First, selection bias may have occurred as the study sample has slightly more rural EDs than the national average, although in the analyses no substantial differences between the different types and locations of hospitals were encountered regarding the organization and staffing of EDs. Second, although a follow-up interview was conducted after the online survey and a stable interviewer was present at all interviews, there were various types of respondents, yielding potentially inter-interview variability in responses. Additionally, the survey utilized was newly created without the benefit of (previous) validation. Third, the QRF does not set minimum standards for outcome or process performance indicators, nor does it measure the actual performance of EDs. These elements are often a part of other initiatives intended to improve the quality of EM care [31].

Fourth, the optimal number and choice of CCAs can be debated. Notably, this was discussed as part of the baseline measurement; the ED professionals and the expert panel agreed on these six CCAs, but the question of which CCAs to include remains the subject of ongoing discussion. In December 2009, a directive regarding obstetrics was published in The Netherlands. The directive establishes minimum quality requirements for hospitals regarding 24/7 availability of obstetrics and pediatricians [32]. Thus, childbirth is an example of an item that has potential as a CCA that may be a valuable addition to this QRF. Last, according to the requirements of the QRF, some CCAs may be considered as (too) limited. For instance, adding neurosurgical capability to the CCA regarding CVA would be considered desirable by many experts.

## Conclusion

This study showed that Dutch EDs fall short of what the expert consensus panelists considered minimum prerequisites for adequate EM care. The process of systematic enquiry revealed this information for the first time, resulting in the implementation of a QRF for Dutch ED personnel that is intended to improve the quality of EM care. Although further testing is needed following implementation to document its effectiveness, this model, as well as the specific process involved in setting up the QRF, could be useful for other countries that face similar EM situations, i.e., that have limited or no standards for ED personnel.

## Endnotes

^a^As trauma care already has its own quality requirements regulated via the Dutch association of Trauma centers, this was left out of the quality requirement framework and is subsequently not part of this paper.

^b^ The main stakeholders involved are: The Dutch Association of Ambulances (RAV), The Dutch Association of Academic Hospitals (NFU), The Netherlands Society for Emergency Physicians (NVSHA), V&VN Dutch Nurses’ Association, The Dutch Association of General Practitioners (NHG), The Dutch Association of Hospitals (NVZ), The Dutch Association of Intensive Care medicine (NVIC), The Dutch Association of Medical Specialists (OMS), The Dutch Association of Trauma Centers (LVTC) and The Ministry of Health (MinVWS).

^c^Hospitals can self-determine the length, content and manner of training (skills practice vs. lecture vs. cases) in any way an individual hospital chooses, although efforts are underway to standardize this to some extent.

## Competing interests

The authors declare that they have no competing interests.

## Authors' contributions

DI drafted the manuscript together with HvdP. Both authors read and approved the final manuscript.
